# General practitioner’s clinical practices, difficulties and educational needs to manage Alzheimer’s disease in France: analysis of national telephone-inquiry data

**DOI:** 10.1186/1471-2296-14-81

**Published:** 2013-06-13

**Authors:** Dominique Somme, Arnaud Gautier, Stéphanie Pin, Aline Corvol

**Affiliations:** 1 CHU de Rennes, Service de Gériatrie, Rennes Cedex 9, 35033, France; 2 Université de Rennes 1, Faculté de Médecine, Rennes, 35000, France; 3 Institut National de Prévention et d’Éducation pour la Santé, Saint-Denis Cedex, 93203, France; 4 German Centre for Neurodegenerative Disease (DZNE), Bonn, Germany; 5 Université Paris-Descartes, Éthique Médicale, Paris, France

**Keywords:** Alzheimer’s disease, Opinion inquiry, Educational needs

## Abstract

**Background:**

The literature has emphasized the role of general practitioners (GPs) in caring for Alzheimer’s disease (AD) patients. Within the framework of the French national AD plan, an inquiry was undertaken to identify the clinical practices, difficulties and training needs of GPs managing this pathology.

**Methods:**

A random sample from a representative national listing of continental French GPs following ≥1 AD patients comprised the study population. Participants completed a standard questionnaire on their clinical practices, difficulties and educational needs for AD management. Feeling insufficiently trained was subjected to univariate and multivariate analyses.

**Results:**

A minority of the 974 respondents declared using questionnaires in their diagnostic evaluation (15.2%), told the patient the diagnosis (8.2%) and was aware of the national recommendations for AD (41.9%). Behavioral disorders represented the most common (73.5%) problem encountered, while half of the GPs considered management of comorbidities easy roles to fulfill. In comparison, coordination of care and assistance did not seem to be a primary problem. A score was calculated, attributing 1 point to each of the following items: need for further education in terms of communications with the family, with patients, disclosing the diagnosis, and non-drug treatments. The factors linked to feeling insufficiently trained for 3 or 4 of the 4 items were: female sex; not involved in educational programs (for parents/family and patients) and no activity related to training medical students.

**Conclusions:**

Our study identified gaps in French GP training concerning AD diagnosis practices and diagnosis announcement. GPs seemed aware of their educational needs and described difficulties in managing behavioral disorders. Our findings enabled the definition of policy priorities to provide training and disseminate information.

## Background

In many countries, Alzheimer’s disease (AD) is becoming a public health priority. This progressive degenerative pathology requires long-term therapeutic adaptation, with the patient, his/her entourage and medical–social professionals having to adapt to the disease’s progression and that of the familial and environmental context [1]. The general practitioner (GP) is the principal go-between for patients and families, even if, in France, as in other countries, the initiation of treatment is reserved to specialists and specialized centers [1,2]. The absence of a curative therapy pushes caregivers, non-drug interventions and medical–social services to the forefront, which requires that health professionals have good mastery of local resources to guide the patient and his/her entourage [3,4]. At more advanced stages, communication difficulties limit exchanges during consultations and the patient’s participation in his/her own care [5]. In addition, more than in other chronic diseases, health professionals insist on the importance of aiding families [6]. Often perceived as ‘incapable’ of being actors in their own health because of their cognitive deficits, AD patients frequently have the feeling of being excluded from their care, in favor of the principal caregiver, even at an early disease stage [7].

We conducted an opinion inquiry on AD, via a questionnaire given to a representative population of continental French GPs, with the objectives of describing their practices, and identifying their difficulties and educational needs to manage AD, to prioritize future programs to improve the quality of first-line care delivered to AD patients and those afflicted with associated pathologies.

## Methods

We describe herein the analysis of data derived from a national telephone inquiry, called Health Barometer, conducted every five years by the Institut National de Prévention et d’Éducation pour la Santé (INPES, French National Institute for Health Prevention and Education), which delegates its running to a polling institute (GfK-ISL, Issy-les-Moulineaux, France). Many different topics were broached in this inquiry, such as the screening for viral hepatitis, human immunodeficiency virus or cancers, vaccination practices, addiction management and therapeutic patient education. These inquiries are used to enrich reflections on establishing public health priorities. The target population was GPs in private practice, who followed ≥1 AD patients during the past year. The complete methodology and the whole questionnaire were previously published [8].

### Modality of respondent selection

The computerized database of physicians’ addresses, provided by the Cegedim® Company (Boulogne-Billancourt, France), comprises doctors’ addresses, phone numbers and e-mail addresses collected primarily by pharmaceutical companies’ representatives (17,000 representatives update their continental French client lists daily). This database contains information on 55,772 GPs in private practice, and is the most reliable and updated French GP database. All the randomly selected French GPs were informed of this inquiry and its objectives by a joint letter sent by the INPES and the Advisory Board of the National Order of French Physicians.

The goal was to obtain a sample of about 2,000 interviews, i.e., slightly more than 1 per 30 GPs. To achieve this number and taking into account those that could not be contacted, 3,892 telephone numbers were extracted. To assure a reasonable questionnaire length, all GPs did not receive all the modules, with the AD-module questions being posed only to about half the GPs selected randomly.

In the context of clearly diminished response rates of the general population and health professionals to inquiries, the variety of procedures offered to limit participation refusal (see below) enabled us to obtain an acceptable participation rate.

All the interviewers underwent training specific to using the questionnaire. The daily presence of an INPES representative with expertise in this type of inquiry, who had also participated in writing the study protocol, assured its supervision.

Every phone number was called 20 times, and the interviewer let it ring 5 times. The number was redialed 10 minutes later when the line was busy and 3 hours later when there had been no answer. Each GP could respond immediately or make an appointment at a future time and date of his/her choice. During the interview, if the respondent was interrupted, an appointment to complete the questionnaire was proposed. In addition, using the Internet as a means of complementary questioning countered the two main arguments given for refusing to participate: the telephone-interview method for the initial inquiry and the lack of time. The online questionnaire could be filled out outside of office hours at any time, notably at times and on days that the telephone inquiry could not be conducted. This procedure collected 185 additional completed questionnaires, representing 9% of the entire sample and 10% of first-line refusers. GPs participating fully in the inquiry were compensated, receiving 30€ from INPES, which represents 1.5 × the nationally standardized consultation fee.

### Inquiry duration

The inquiry was conducted from 6 November to 31 January 2009 (11 weeks). The computer-assisted telephone interview lasted a mean total of 25 minutes. The online inquiry was offered to every GP who refused the telephone interview or who failed to honor the appointment made for him/her to respond to the questionnaire items.

### GPs’ characteristics

Certain characteristics, multiple choice responses self-declared by the GPs, were subsequently used as explanatory variables: age; sex; type of practice (single or group); activity sector defined by partial or total reimbursement of the consultation fee by the French National Health Insurance; involvement in the education or advanced classes for medical students; number of patients seen per day above the median; experience in setting up educational health programs for the public; regular or systematic practice of alternative medicine techniques (e.g., homeopathy, mesotherapy, acupuncture…); >10% of practice patients benefitting from universal health-insurance coverage (a program specifically dedicated to improving access to care of the most underprivileged individuals); participation in a health network (type of organization favoring links between private practice and hospitals); and the number of days/week of presence in the office (5–7 days/week or less).

### The AD module

This questionnaire (see Additional file 1) was created by a working group composed of experts in public health, GPs, specialists (neurologists, geriatricians…), representatives of family associations and institutions. The themes retained for it were defined according to the Delphi method [9,10].

The questions were presented according to theme. The first item concerned professional activities: the number of AD patients among the GP’s clientele. The second referred to the doctor’s clinical practices (e.g., the use of tests and the frequency of disclosing the diagnosis to the patient and his/her entourage). The third concerned his/her difficulties in relationship with patients or in the management of different aspects of AD (e.g., comorbidities, coordination of care, information about available social assistance and the management of behavioral disorders). The fourth and last item was the GP’s self-perception of his/her level of training concerning certain aspects of managing AD patients: communication with the patient, communication with the family, announcing the diagnosis and non-drug treatments of the disease.

A simple additive score was devised to evaluate how these GPs self-estimated their educational level to manage AD. The score ranges from 0: considered insufficient for all facets of AD; to 4: adequately trained to handle all of them.

### Ethical issues

This study did not fall within the scope of the Helsinki Declaration, since it was not “medical research involving human subjects” but an anonymous opinion survey of GP’s concerning their practices and training needs. This study was conducted in accordance with the recommendations of the National Commission for Data Protection and Liberties (CNIL France) that approved it (authorization no. 112444).

### Statistical analyses

Descriptive results are expressed as numbers (%) for categorical parameters and means ± standard deviation (SD) for continuous data. Univariate analyses of continuous variables used Student’s *t*-test, while qualitative variables were compared with the chi-square test. Significance was defined as *p* = 0.05.

The score assessing self-perceived training needs, derived from the analysis reported herein, was tested using Cronbach’s α-coefficient to verify its internal consistency.

For multivariate analyses, we used a binary logistic regression. In one first logistic regression analysis the dependent variable was to feel insufficiently trained for any 1 or more themes. Secondly, in a series of multivariate analysis, the dependant variable were respectively (all dimensions considered tested independently) : use of diagnostic tests; diagnosis disclosure; referral to a specialist; patients/families orientation towards assistance or home care; awareness of the national AD recommendations; comorbidities management; easy coordination of care; easy management of behavioral disorders. The independent variables were the same for both and were all the available characteristics of the interviewed physician. The results obtained are expressed as odds ratios (OR) [95% confidence interval (CI)]. All statistical analyses were computed with Stata V10.SE software. We used Hosmer Lesmeshow test and R^2^ Nagelkerke as quality indicator of our multivariate analyses.

## Results

### Population characteristics

During the study period, among the 3,892 telephone numbers dialed, 245 (6.3%) were ineligible (wrong numbers, specialist, retired, on prolonged leave…), 1,125 (28.9%) refused to participate in the interview, 357 (9.2%) did not keep the appointment agreed upon or complete the online questionnaire, and 82 (2.1%) stopped in the middle of the questionnaire. Notably, 2,083 (53.5%) completed the questionnaire. Sociodemographic findings were compared to known national statistical data, which confirmed that the sample was representative (Table 1). The frequencies observed for the entire responding population were very similar to those provided by diverse administrative sources concerning the distributions of sex, age, region or activity sector (partial or total fee reimbursement). This similarity of results suggests that our sample adequately represented the national GP population, which led to not adjusting data for these criteria when presenting our findings.

**Table 1:** Representativeness of the sample: comparison of study-population values to national administrative databases

	**Inquiry respondents**					
**Sample characteristic**	**Phone**	**Internet**	**Total**	**AD module**	**ADELI**^**a **^**2008**	**SNIR**^**b **^**2008**
*N*	1,898	185	2,083	1058	68,313	61,359
Sex (%)						
Male	69.0	77.8	69.8	69.1	69.4	72.0
Female	31.0	22.2	30.2	30.9	30.6	28.0
Age (%)						
<40 years	13.0	8.6	12.6	11.9	14.8	10.5
40–49 years	29.2	28.1	29.1	29.8	30.3	28.5
50–59 years	43.6	50.3	44.2	44.7	42.7	45.0
>59 years	14.2	13.0	14.0	13.6	12.2	15.9
Practice region^c^ (%)						
Île-de-France	14.1	14.1	14.1	14.0	17.7	16.4
Northwest	17.1	18.4	17.2	17.8	18.3	18.6
Northeast	23.7	25.4	23.9	23.8	22.1	22.5
Southeast	30.6	24.3	30.0	30.1	26.7	27.2
Southwest	14.5	17.8	14.8	14.3	15.2	15.3

Values are expressed as percentages unless stated otherwise.

^a^ADELI (Automatisation DEs Listes: Automation of the Lists) is a national database that records all practicing professionals who must register their education degrees with State authorities. Included herein are only the GPs declaring at least half of their professional activity in private practice during 2008.

^b^SNIR (Système National Inter-Régimes: National System between Reimbursement Regimens) is an information database, managed by the health-insurance organizations, that collects all the professionals whose private-practice activities earned a reimbursement during the previous year. The SNIR registry includes, in addition to doctors in private practice, full-time hospital staff physicians with private consultations in the hospital.

^c^Locations where the GPs exercised their profession were apportioned according to regional telephone codes.

From that sample, 1,058 GPs were randomly selected to complete the AD module; no significant difference distinguished their characteristics from those of the other GPs. Among them, 974 (92.1%) followed ≥1 AD patients and comprised the study population, whose participants were distributed throughout continental France, and 324 (33.3%) of them declared having managed >10 AD patients during the preceding year (Table 2). Physicians not included because they declared no AD patients were more often women, with a lower patient load and part-time activity, more often practicing alternative medicine and more of them received only partial fee reimbursement.

**Table 2:** Characteristics of general practitioners (GPs) interviewed: comparisons according to their Alzheimer’s disease (AD) patient load

	**GPs interviewed for the**		**GPs following**		
**Characteristic**	**Inquiry**	**AD module**	**≥1 AD patients**	**No AD patient**	***p***
*N*	2083	1058	974	84	
Mean age	50.6	50.7	50.9	47.7	0.0007
Age >50 years	53.9	54	55.1	40.5	0.010
Male	68.8	69.1	70.7	50.0	<0.0001
Type of office (alone vs group practice)	48.0	47.7	47.2	54.2	0.217
Total (vs. partial) fee reimbursement	89.1	88.5	89.6	75.0	<0.0001
Role as an educator (medical students)	20.4	20.1	20.6	14.3	0.164
High patient load (>21 patients/day)	59.8	59.6	62.1	31.3	<0.0001
Involved in educating the public	33.6	33.1	33.4	29.8	0.500
Alternative medicine techniques (used often or always)	23.8	24.0	22.1	46.4	<0.0001
>10% of clientele underprivileged	25.6	26.6	27	22.2	0.353
Participation in a network	38.9	38.8	39.6	28.6	0.046
5–7 days/week (vs. fewer days) in office	58	57.7	59.4	37.8	<0.0001

Values are expressed as percentages unless stated otherwise.

### Declared practices of the interviewed GPs

Among GPs managing AD patients, 148 (15.2%) used standard questionnaires to evaluate memory and cognitive disorders. Although 688 (70.6%) systematically announced the diagnosis to the patient’s family, only 80 (8.2%) told the patient. Indeed, 310 (31.8%) never disclosed the diagnosis to the patient. Male and younger GPs declared more often that they “usually” or “always” disclosed the diagnosis to the patient (Table 3). Notably, 566 (58.1%) of the interviewed GPs admitted not being aware that national recommendations existed. Once patients were identified, 788 (81.2%) systematically referred them to a specialist. Systematic referral was associated with the GPs older age and higher patient load. In addition, 423 (43.5%) always or frequently indicated the names of social or home-care services to patients and/or family.

**Table 3:** Logistic-regression multivariate analyses of the declared practices or difficulties according to general practitioners’ characteristics

	**Dependent variables**							
**Independent variables**	**AD diagnostic tests used**	**Disclosed AD diagnosis to patient**	**Systematic referral to specialist**	**Referred to social services**	**Aware of national guidelines**	**Difficulties: easy management of**		
						**Comorbidities**	**Coordination of assistance**	**Behavioral disorders**
*N*	925	925	925	925	925	925	925	925
Age >51 yr	1.15 (0.78–1.72)	0.61† (0.44–0.84)	1.66† (1.15–2.39)	1.49† (1.11–2.00)	1.19 (0.89–1.59)	0.95 (0.70–1.30)	1.04 (0.78–1.38)	0.93 (0.68–1.29)
Male	0.75 (0.49–1.12)	1.57* (1.09–2.26)	0.66* (0.44–0.99)	0.47‡ (0.34–0.64)	1.43* (1.04–1.97)	1.66† (1.20–2.29)	0.92 (0.68–1.25)	1.22 (0.86–1.75)
Type of practice (alone vs. group)	0.97 (0.66–1.43)	1.11 (0.81–1.53)	0.77 (0.53–1.10)	1.28 (0.96–1.70)	1.10 (0.82–1.46)	0.99 (0.73–1.34)	1.14 (0.87–1.51)	1.24 (0.90–1.70)
Total (vs. partial) fee reimbursement	1.66 (0.82–3.38)	1.31 (0.76–2.27)	0.95 (0.53–1.70)	0.91 (0.57–1.43)	1.46 (0.91–2.34)	1.24 (0.77–1.99)	1.20 (0.77–1.87)	0.88 (0.53–1.46)
Role as an educator (medical students)	0.87 (0.54–1.39)	1.29 (0.89–1.85)	0.52† (0.35–0.78)	0.99 (0.71–1.39)	1.30 (0.93–1.81)	0.80 (0.56–1.13)	1.14 (0.82–1.59)	0.92 (0.63–1.34)
High patient load (>21/day)	0.83 (0.57–1.22)	1.28 (0.92–1.77)	1.51* (1.06–2.162)	1.06 (0.80–1.42)	1.01 (0.76–1.36)	0.98 (0.72–1.32)	1.30 (0.98–1.72)	1.18 (0.85–1.63)
5–7 days/wk (vs. fewer days) in office	1.20 (0.81–1.79)	1.08 (0.78–1.50)	0.93 (0.65–1.35)	0.85 (0.64–1.14)	0.91 (0.68–1.21)	1.19 (0.88–1.61)	1.14 (0.86–1.51)	1.12 (0.81–1.54)
Involved in educating the public	0.91 (0.61–1.35)	1.30 (0.94–1.78)	1.22 (0.84–1.78)	1.19 (0.89–1.59)	1.70‡ (1.28–2.27)	1.35 (0.99–1.83)	1.08 (0.82–1.44)	1.67† (1.22–2.28)
Alternative medicine techniques (often or always)	0.73 (0.45–1.19)	1.18 (0.82–1.70)	1.34 (0.87–2.09)	1.08 (0.78–1.50)	0.89 (0.64–1.25)	0.98 (0.69–1.40)	1.04 (0.75–1.43)	0.93 (0.64–1.35)
>10% of clientele underprivileged	0.92 (0.62–1.40)	1.00 (0.71–1.40)	1.00 (0.68–1.48)	0.92 (0.68–1.25)	0.88 (0.65–1.20)	0.85 (0.62–1.16)	0.79 (0.59–1.06)	1.03 (0.73–1.44)
Participating in a network	0.99 (0.68–1.45)	1.10 (0.81–1.50)	0.91 (0.64–1.29)	1.49† (1.13–1.96)	1.52† (1.15–2.01)	1.10 (0.82–1.48)	1.12 (0.86–1.47)	1.32 (0.98–1.80)
R^2^ Nagelkerke	0.018	0.041	0.047	0.061	0.059	0.030	0.015	0.032
Hosmer Lesmeshow test (sig.)	0.121	0.757	0.533	0.275	0.745	0.668	0.139	0.999

*p < 0.05; †*p* < 0.01; ‡*p* < 0.001.

Values are expressed as odds ratios (95% CI), unless stated otherwise. Multivariate analyses used information from 925 GPs for whom no data were missing.

### GPs’ difficulties with AD

When they were questioned about the quality of their relationships with AD patients, 754 (77.4%) felt comfortable in their role (completely at ease: 191 (19.6%), or quite at ease: 563, 57.8%).

Asked about the degree of difficultly encountered in managing certain aspects of accompanying AD patients, 664 (68.2%) felt completely or mostly at ease managing their comorbidities, 506 (52.0%) easily handled the coordination of care and assistance completely or mostly, and 538 (55.2%) were completely or mostly at ease providing information on available social assistance. In contrast, 716 (73.5%) considered the associated behavioral disorders difficult to manage.

### GPs’ self-perception of their training-adequacy level

The majority of GPs interviewed considered their training sufficient concerning communications with the families (781, 80.2%), their patients (653, 67.0%), announcing the diagnosis (584, 60.0%) and the non-drug treatments for AD (502, 51.5%). The score (range: 0–4; mean: 2.6 ± 1.4) evaluating the need for further education, calculated on the 4 preceding items, achieved a Cronbach α of 0.75. The score distribution showed that 348 (35.7%) of the interviewed GPs felt adequately trained to handle all 4 dimensions, 256 (26.3%) for 3 items, 1212 (12.4%) for 2 topics and 118 (12.1%) for only 1. Finally, 131 (13.5%) felt insufficiently trained for the 4 dimensions.

According to our multivariate analyses (Table 4), female GPs (OR: 1.44 [95% CI: 1.02–2.03]) and those not involved education sessions for the public (OR: 1.44 [1.03–2.02]) had a higher probability of estimating themselves insufficiently trained for 3 or all 4 dimensions considered.

**Table 4:** **Logistic-regression multivariate analyses**^**a **^**of feeling insufficiently trained (dependent variable) according to general practitioners’ characteristics**

**Independent variables**	**Odds ratio**	**95% CI**	***p***
Age >50 years	0.72	0.52–1.00	0.053
Female	1.44	1.02–2.03	0.036
Type of office (alone vs group practice)	0.94	0.68–1.30	0.702
Total (vs partial) fee reimbursement	1.38	0.79–2.40	0.254
Role as an educator (medical students)	0.74	0.49–1.11	0.144
High patient load (>21 patients/day)	0.77	0.56–1.07	0.115
5–7 days/week (vs fewer days) in office	0.98	0.70–1.36	0.894
Involved in educating the public	1.44	1.03–2.02	0.033
Type of practice (individual or group)	1.16	0.80–1.68	0.437
>10% of clientele underprivileged	1.09	0.78–1.53	0.617
Participation in a network	1.04	0.76–1.42	0.789

^a^Multivariate analysis was based on 925 GPs for whom no data were missing.

R^2^ Nagelkerke: 0.032; Hosmer-Lemeshow test : p = 0.339.

### Association between GPs’ characteristics, declared practices, difficulties and self-perceived training needs

The influence of GPs’ characteristics on their declared practices, their knowledge of the national recommendations and their encountered difficulties was studied with multivariate analyses whose results are reported in Table 3. Most of the GPs’ characteristics did not show any statistical association with clinical practice or difficulties. We only noticed a significant association between male sex and self-sufficiency (less referral to specialist and social services) and self-assessment of their practice (more disclosure and more prone to declare to be aware of national guidelines, and to declare an easy management of comorbidities). The age of the GP also influenced practices: the older GPs tend to declare less disclosure of the diagnosis but more referral to a specialist or social services. The characteristics “GPs having a role as an educator” and “GPs with high patients load” have only a small negative influence on the probability of systematic referral to specialized care. GPs who are involved in educating the public declared more frequently being aware of national guidelines (but without any statistical difference in disclosing the diagnosis), and declared less difficulties in managing behavioral disorders. Finally, GPs participating in a network declared more frequently referring to social services and being aware of national guidelines.

## Discussion

GPs are core players in the accompaniment of AD patients and their entourage [1,11,12]. Nevertheless, to our knowledge, no nationwide inquiry of equal size had been conducted previously on a random sample representative of the GP population. As shown by our results, 9/10 participants had managed ≥1 AD patients during the preceding year and a third of them followed >10.

### Clinical practices

Although the French national public health authorities, in accordance with international experts [1], recommend using standardized diagnostic tests to diagnose AD, only 15.2% of our respondents used such tools. That percentage is quite close to the 19% reported in a regional study conducted in France in 1994 [13] but much lower than a voluntary investigation that queried physicians participating in a public health network [14]. That all the approached doctors were GPs in France who were asked participate but could refuse to do so, as opposed to being volunteers who spontaneously proposed their participation, probably explains that difference in the representativeness of the sample questioned. Kaduszkiewicz et al.’s study has shown that less use of diagnostic tests was associated with the feeling of being insufficiently trained [15].

Our test-usage rate could also reflect previous statements indicating that the Mini-Mental State Examination (MMSE) is also deemed poorly reliable overall by GPs [16]. Other more rapid tests that current guidelines recommend are still not widely disseminated [14]. This finding should also be viewed in the context of the time constraints during office consultations [3,4]. Thus, more widespread diffusion of the recommended rapid cognition tests (5-word test, verbal fluency test, clock test, 7-minute test, etc.…) seems desirable. Acceptance of these tests by primary-care physicians should be a research priority, to finally retain one or several tests the most compatible with their medical practices.

A national guideline recommends revealing the diagnosis to the patient using a step-by-step-disclosure approach of the representations, and wishes of the person and his/her entourage. Although this inquiry was unable to evaluate the GPs’ use of such an approach, its findings confirmed the application of different (somewhat heterogeneous) practices for disclosing the diagnosis: the GPs preferred addressing the patient’s entourage first and confronting the patient only secondarily, as previously reported [14,17,18]. Moreover, the diagnosis-disclosure rates were much lower than those reported in earlier studies based on fewer participants from a network of GPs [14].

Although several differing opinions exist concerning diagnosis disclosure [19], the results of the great majority of the studies demonstrated that the affected individuals were largely in favor of being told [20-24], whereas the practices reported herein reveal a low rate of telling the patient and a slightly higher percentage of announcing it to the family. The French guidelines for patients with Alzheimer’s disease, addressed to all medical practitioners, including family physicians, considered the diagnosis disclosure as an indicator of the quality of care. It could be debatable considering that most of the literature [17-24] on this subject examines the perspectives of patients or specialists, but very little is known about family physicians’ perspectives.

The reasons why GPs do not disclose the AD diagnosis to patients (and sometimes even the family) were linked to the physician’s own projections of the distress associated with that announcement or, in contrast, to its incomprehensibility [25] with social representations, notably the resulting stigmatization [26-28], but also therapeutic nihilism [25,29] and, finally, the lack of sufficient training [3,13,29]. Conditions facilitating diagnosis disclosure were recently analyzed, based on a literature review, and the opinions of patients and professionals. New research will be necessary to specify the GP’s role in making this announcement [30].

### Orientation for assistance and awareness of national recommendations

The national recommendations were diffused by large-scale postal mailing of the printed document. This means of disseminating information is simple and inexpensive but its efficacy is routinely questioned [31]. Indeed, that 58.1% of the GPs reported being unaware of those guidelines represents one of the worst information-penetration levels [32-34].

French GPs have high referral rates (81.2% called upon a specialist vs. only two-thirds of those questioned by Wilcox et al. [35]). Indeed, in France, the gatekeeping role of primary-care physicians, in terms of second-line practitioners, is limited. Patients and their families can decide, subjected to a minor reimbursement penalty, to consult directly with a specialist. This particular organization, with neurologists in private practice, led experts to qualify our system as competitive [36] and, in their opinion, could make GPs reluctant to refer their patients. Thus, the high observed referral rate could indicate a low degree of competition concerning dementia patients.

The lack of GPs’ information on available social services, judged in other contexts as the major impedance to their good use [4], does not seem, for our study, to explain the insufficient referral to these services. Some GPs considered themselves sufficiently informed but still had trouble organizing medical–social assistance services.

Even though the motivations for referral are probably multifactorial [26], it is likely that this practice contributes to a good level of care for the patients. In an earlier French study, only 19.7% of the AD patients 80 years old were referred to a specialist [37]. Two plausible and perhaps associated explanations can be advanced: an increased referral rate to a specialist between the Three-City Study inquiry period (1999–2004) and 2008, and GPs’ overestimations of their referral rates.

Turning towards medical–social services was less frequent (43.5%) but, without individual data on the patients referred, it is difficult to know if it is a sign of good or difficult access to assistance.

### GPs’ difficulties

Behavioral disorders represented the most common (73.5%) problem encountered, while about half of the GPs considered management of assistance or comorbidities easy to fulfill roles. These observations are consistent with those of a recent, more detailed, French study on a smaller sample [38]. The difficulties of managing behavior disorders and the multiple medications were previously found in studies on small populations [3,29], and, notably, was proposed as one of the factors explaining the GPs’ impression of insufficient competence. Medical–social services’ difficulties in maintaining patients at home was also described [3,4,27] as a hindrance for GPs, but less so than the behavioral disorders or comorbidities, as we found.

### Continuing education

The majority of studies on GPs’ knowledge of AD concerned their ability to detect symptoms and make the diagnosis (especially early) or even their mastery of the drugs used [39-41]. The need to improve GPs’ communication skills concerning AD was emphasized previously [39,40,42]. In addition, even though non-drug therapies have been highlighted as being an integral part of the therapeutic strategy [1], with a role for the GP in their monitoring, the need for continuing education in this field does not seem to have been assessed either.

Sociodemographic criteria, like age or sex, affected the GPs’ practices and difficulties, as previously described [3,18]. In particular, a gender bias in self-estimated competence, with female physicians according themselves lower competence, was previously described [42]. The other found associations are statistically small and quite intuitive (more referral to social services for GPs with an activity in a network involving social services, more declared knowledge of national guideline in GPs declaring having a educating role, less referral in GPs declaring having a certain expertise because of their teaching position or their clinical experience).

Notably, concerning non-drug treatments, half of the GPs interviewed considered themselves insufficiently trained. This finding should be viewed in the context of the rather low-level use of non-drug strategies in France and Europe, compared to drug use [39,40].

### Limitations

One of the main limitations of this inquiry concerned the small number of questions it was possible to ask during an interview aimed at achieving a large sample size and covering several health topics. It led the study’s Scientific Committee to select the questions to be asked. Other themes could have undoubtedly been treated and would have identified other practices, difficulties and/or needs for further education. Nonetheless, we think that the selected questions have improved our knowledge of the problems facing GPs, compared to previous publications.

Among its limitations, this inquiry was conducted before the actual announcement and support foreseen in the national Alzheimer Plan [43]. Its different steps, experimental or not, will undoubtedly affect the perceptions and knowledge of GPs concerning their role and practices. Repeating the inquiry with GPs at the end of the Alzheimer Plan would enable an evaluation of these changes and an appreciation of the impact of this governmental program. Given the importance of the system’s organization on the clinical practices and knowledge of doctors, generalization of our results seems difficult outside of France, without taking certain precautions. Nevertheless, the similarity of the recent IMPACT inquiry on the practices of doctors in Europe seems to suggest that our observations are probably valid in other European settings [39].

## Conclusion

In conclusion, GPs are central actors in the quality of care delivered to AD patients. Our study was able to collect the opinions of a large representative sample of French GPs. The difficulties that they encounter notably linked to AD-associated behavioral disorders, and the need that they expressed for greater access to continuing education, especially on non-drug management of these disorders, enabled establishment of priority avenues to pursue to provide training and disseminate information. The relationship between education and improved quality of care delivered (especially in disclosing the diagnosis and patient involvement in the care plan) should be the object of future specific investigations.

## Abbreviations

GP: General practitioner; AD: Alzheimer’s disease; OR: Odds ratios; CI: Confidence interval

## Competing interests

The authors have no conflicts of interest to declare.

## Authors’ contributions

DS participated in the design of the statistical analysis plan, interpretation of the data, and drafted and revised the manuscript. AG participated in the design of the study, performed statistical analysis, and revised the final version of the manuscript. SP conceived of the study and participated in its design and coordination, and revised for the final version of the manuscript. AC helped interpret the data, draft the manuscript and revised for the final version of the manuscript. All authors read and approved the final manuscript.

## Pre-publication history

The pre-publication history for this paper can be accessed here:

http://www.biomedcentral.com/1471-2296/14/81/prepub

## Supplementary Material

Additional file 1 Alzheimer’s Disease Module (Microsoft Word® format). Click here for file

## Prior presentations

The methodology was previously presented at the VIième Colloque Francophone sur les Sondages, 22–25 March 2010, Tangiers, Morocco.
