# Supplementary material for: General practitioner’s clinical practices, difficulties and educational needs to manage Alzheimer’s disease in France: analysis of national telephone-inquiry data
Source: BMC Fam Pract. 2013 Jun 13;14:81. doi: 10.1186/1471-2296-14-81 (PMC3682915; doi:10.1186/1471-2296-14-81)
Supplement: Additional file 1 — Alzheimer’s Disease Module (Microsoft Word® format). [file 1471-2296-14-81-S1.doc]

Alzheimer’s Disease Module

Let’s go to another subject, we are going to talk now about your patients suffering from Alzheimer’s disease and related disorders.

Q 1 In the last twelve months, how many of your patients suffered from Alzheimer’s disease?

1 none

2 from 1 to 4

3 from 5 to 9

4 ten and over

5 no idea

6 don’t want to answer this question

If the answer was > 1 : the questionnaire continue

Q2 In a general way, do you announce the Alzheimer’s disease diagnosis to the patient himself?

Q3 In a general way, do you announce the Alzheimer’s disease diagnosis to the patient’s family?

Q4 Do you refer your patients with Alzheimer’s disease to a specialist?

Q5 Do you refer your patients with Alzheimer’s disease to a coordination centre for social services?

For Q2 to 5, possible answers were:

1 systematically

2 often

3 sometimes

4 never

5 no answer

Q6 I am going to mention you some characteristics of the care for patients with Alzheimer’s disease; you, yourself, how would you grade your level of easiness with this aspects:

a: management of behavioural troubles

b: management of coordination of assistance (health and social care)

c: management of comorbidities

For Q6 a to c, possible answers were:

1 very easy

2 somewhat easy

3 somewhat uneasy

4 not easy at all

5 no answer

Q7 In your relationship with your patients with Alzheimer’s disease,how do you feel?

1 very comfortable

2 somewhat comfortable

3 somewhat uncomfortable

4 not comfortable at all

5 no answer

Q8 Considering your patient suffering from Alzheimer’s disease and related disorders, do you feel yourself sufficiently trained for

a. diagnosis disclosure

b. patients communication

c. family communication

d. non-pharmacological approaches (as cognitive stimulation, physiotherapy, orthophony, etc…)

For Q8 a to d possible answers were

1 yes

2 no

3 no answer

Q9 In June 2008, the Health National Authority published recommendations about diagnosis and care of patients suffering from Alzheimer’s disease. Do you know these recommendations?

1 yes

2 no

3 no answer
